# Ongoing maturation in the time-compressed speech test

**DOI:** 10.6061/clinics/2018/e407

**Published:** 2018-11-16

**Authors:** Camila Maia Rabelo, Caroline Nunes Rocha-Muniz, Eliane Schochat

**Affiliations:** Fisioterapia, Fonoaudiologia e Terapia Ocupacional, Faculdade de Medicina FMUSP, Universidade de Sao Paulo, Sao Paulo, SP, BR

**Keywords:** Hearing, Auditory Perception, Auditory Pathways

## Abstract

**OBJECTIVES:**

To verify the neuromaturational influence in the ability of auditory closure, that is, to verify the performance of children and young adults in the ability of auditory closure, through the time compressed speech test (TCS).

**METHODS:**

Thirty children (8 to 10 years old) and 30 young adults (16 to 24 years old) with normal hearing without complaints (neurological, cognitive, auditory processing) who performed TFC (monosyllables and disyllables) with a compression ratio of 60% in both ears. Statistical analysis was performed using analysis of variance (ANOVA) and ANOVA with repeated measures with a significance level of 0.05. The minimum statistical power was 80%.

**RESULTS:**

In the comparison between ears, there was no significant difference between groups for the monosyllables. For disyllables, the second ear tested was better in children, and the right ear was better than the left ear for young adults. In the comparison between modalities (monosyllables and disyllables), children did not show significant differences. The performance of the young adults was better in the disyllables in both ears. Comparing the age groups, the young adults were better than the children for both modalities and ears.

**CONCLUSION:**

The study has demonstrated the influence and impact of age (maturational factor) on TCS test performance, showing the importance of establishing normality patterns for various age groups to provide a standardized tool for evaluation of auditory closure ability.

## INTRODUCTION

Low-redundancy monotic speech tests are administered monaurally with degraded speech stimuli. One of the most frequently used low-redundancy monotic tests is the time-compressed speech test (TCST), which involves compression of the speech stimulus by removing portions of the sound wave electromechanically and then joining the remaining segments and presenting them at normal speed. This compression reduces redundancies extrinsic to speech, allowing the duration to be reduced by changing the speed without changing the fundamental frequency of the signal 1,2.

The TCST is mainly used to explore aspects of auditory closure, wherein individuals with normal hearing and intact extrinsic and intrinsic abilities can easily perform the closure, correctly processing the stimuli, even with the loss of some cues. The test has also been used to evaluate temporal processing 3 and the effects of auditory training.

In addition to the compression effect, speech signal processing can be affected by other factors. Age also affects an individual's performance, with the subject's increasing age improving performance (from childhood to adolescence). In addition, it has been observed that a decline in speech intelligibility does not occur in any studied age group until compression reaches 60% 4-7.

When a large proportion of the acoustic signal is missing, the test becomes cognitive because the individual must use top-down skills to infer the complete message; this process is affected by maturation of the central auditory nervous system 8. Therefore, the TCST can be used to investigate neuromaturation.

The aim of this study was to investigate the effect of neuromaturation on auditory closure ability, that is, to compare children's and young adults' auditory closure performance levels using a time-compressed speech test.

## MATERIALS AND METHODS

This study was approved by the Research Project Analysis Ethics Committee (CAPPesq) (protocol 649/01). The study was conducted in the Laboratório de Neuroaudiologia do Departamento de Fisioterapia, Fonoaudiologia e Terapia Ocupacional da Faculdade de Medicina da Universidade de Sao Paulo.

A total of 30 children aged 8 to 10 years (mean±standard deviation: 9±0.83 years) and 30 young adults aged 16 to 24 years (21.03±2.14 years) participated in the study. The 60 subjects had normal peripheral hearing and good school performance and were without neurological, psychiatric, cognitive or central auditory processing deficits. All children were from public or private schools in the western region of São Paulo. Adults were graduated students or volunteers, together they formed a convenience sample for the study.

After giving their authorization for the study by signing a free and informed consent form, the guardians or individuals themselves (adolescents) completed an anamnesis that listed the main complaints relating to auditory processing changes.

The TCST evaluation was performed bilaterally, with monosyllables and disyllables, with a compression rate of 60%. The test consisted of 25 lists of monosyllabic and disyllabic words compressed to 60% using an electromechanical time compression method 7. The stimulus was monaural and 40 dB above the speech recognition threshold (SRT). The guidance given to individuals was to repeat what they heard, encouraging them to respond even if they were not sure of the answer. Hits and misses were scored in terms of the respective percentages for each ear.

First, the right ear was evaluated, and then the left ear was evaluated because previous studies 7 did not find a statistically significant difference between the first and second ear tested. The same was considered for the test presentation with monosyllables or disyllables; the patients started by one of the two tests randomly because the same study 7 did not demonstrate any influence of stimuli presentation.

Pearson's correlation and linear regression were calculated to determine the strength of the association between age and performance in the TCST for both modalities and both ears. Analysis of variance (ANOVA) and repeated measures ANOVA were applied with a significance level of 0.05. The minimum statistical power was 80%.

## RESULTS

### Difference between the ears (laterality)

**Figure 1:**
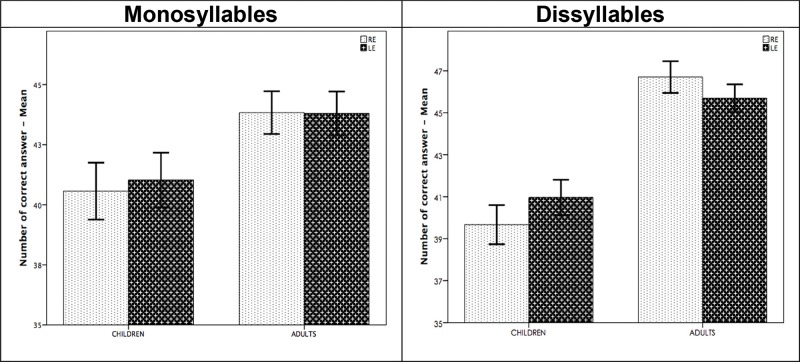
Mean performance obtained between right and left ears without TCS in children and adults groups.

In both the child and young adult groups, comparisons between ears (Figure 1) revealed no significant differences in the TCST in the ‘monosyllable' modality. In the ‘disyllable' test, there was a significant improvement in the performance of the second ear tested (left ear) in the child group (*p*=0.006). The young adult group performed significantly better in the right ear than in the left ear (*p*=0.05).

### Difference between modalities (monosyllable *vs.* disyllable)

**Figure 2:**
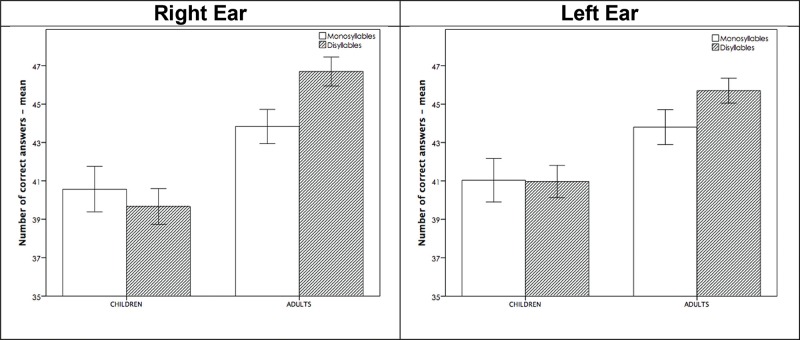
Mean performance among the monosyllable and disyllabic modalities in TCS, in both ears, in the groups of children and adults.

Comparison of the ‘monosyllable' and ‘disyllable' tests (Figure 2) in the child group revealed no significant differences between monosyllabic and disyllabic performance. In contrast, in the young adult group, ‘disyllable' performance was significantly higher than ‘monosyllable' performance for both the right ear (*p*<0.001) and the left ear (*p*<0.001).

### Difference between age groups (children *vs.* young adults)

In the comparison between groups (Table 1), the young adult group performed significantly better than the child group in both the ‘monosyllable' and ‘disyllable' modalities and in both ears.

Table 2 shows the Pearson correlations between age and performance in the TCST for both modalities and both ears. Age was a strong predictor of TCST performance in the disyllable modality, and a moderate correlation was observed in the monosyllable modality.

Linear regression analysis revealed that the ‘disyllable' modality test showed the greatest age effect. We found that 64.4% of the variation in right ear performance and 57.7% of the variation in left ear performance was explained by the age variable. In the ‘monosyllable' modality, the effects of the age variable on test performance variation were 23.6% for the right ear and 21.4% for the left ear.

## DISCUSSION

The aim of this study was to use a time-compressed speech test to evaluate the effects of maturation on auditory closure ability. In general, we found that the maturational factor (age) significantly affected the groups' performance on the TCST in both the monosyllable and disyllable modalities.

### Difference between the ears (laterality)

The first finding revealed by our results concerned the different laterality effects observed in the child and young adult groups. In children, we observed better performance in the second tested ear (left ear), whereas for young adults, better performance was observed in the right ear.

This result was not expected, as hemispheric differences are evident in the normal processing of speech sounds presented in a dichotic manner. The classical model, originally proposed by Kimura 9, proposes that the right ear has an advantage over the left in terms of speech sounds because auditory speech stimuli captured by the right ear are directly processed in the left hemisphere (the hemisphere mainly responsible for speech processing) by means of the action of the contralateral pathways. When speech stimuli are captured by the left ear, they are first directed to the right hemisphere (RH) and are later sent via the corpus callosum for processing in the left hemisphere.

Monotic tests, such as the time-compressed speech test, activate the auditory system's ipsilateral and contralateral pathways. This mechanism neutralizes the effect of laterality and promotes a similar performance between the ears 10.

In this study, we believe that the two groups had different mechanisms in terms of the effect of lateralization and that this difference was related to top-down processing.

In the child group, which showed a left ear (second ear) advantage, we can infer that the “test learning effect”, i.e., previous experience in the first tested ear, can explain why this difference occurred.

In the young adult group, the advantage shown by the right ear (first ear) may be related to attentional factors. In other words, the first tested ear (right ear) required greater attention mechanisms in the young adult than the second ear (left ear).

Another hypothesis could be related to possible cortical influences on the processing of monotic tests. Kimura's theory 9 may be applied in this regard.

We know that the advantages shown by either ear may reflect functional differences between hemispheres. This concept has been described in the literature in regard to dichotic tasks but not monotic tasks.

The first applications of tests using “speech in noise” 11 reported deficits in the ear contralateral to cortical lesions. Subsequent studies using the “speech in noise” test have shown deficits contralateral to the hemisphere, with implications in the auditory cortex 12,13. However, the “speech in noise” test is not affected by inter-hemispheric transfer (corpus callosum).

Based on the above studies, one possibility might be that the right ear advantage observed in our young adult group was caused by processing in the left hemisphere, according to Kimura's hypothesis 9.

### Difference between modalities (monosyllable *vs.* disyllable)

Differences between the child and young adult groups were also observed in terms of monosyllabic and disyllabic performance.

The young adult group showed better means in both ears for the disyllable list than the monosyllable list. In other words, this group performed significantly better in the disyllable modality than in the monosyllable modality.

The child group did not demonstrate this better auditory closure ability and temporal resolution performance in the disyllable modality compared with the monosyllable modality.

One explanation for this difference in the response pattern observed between the groups, that is, that only the young adults performed better in one modality than the other, could be that young adults in the disyllable modality were more facilitated by the neuromaturation of top-down factors (i.e., cognition, lexical and semantic processes and other executive functions) than by bottom-up factors (i.e., sensory encoding), which should not have occurred in the case of the children.

It is widely known that both bottom-up and top-down factors work together in the auditory processing of acoustic information. Both factors affect auditory processing input and thus determine a person's ability to understand auditory information.

In the disyllable modality, the number of acoustic cues was higher than in the monosyllable modality, resulting in an increase in the extrinsic redundancies of the stimulus. Therefore, it is assumed that the greater the knowledge acquired in lexical-semantic processes and the greater the maturation of other higher-level cognitive skills, the easier it will be to interpret and close incoming sensory information, and the better the individual's performance will be 14,15.

Taken together, these top-down factors seem decisive in contributing to performance in the disyllable modality only for young adults, as they have maturational development at higher cognitive levels than children.

### Difference between age groups (children *vs.* young adults)

**Figure 3:**
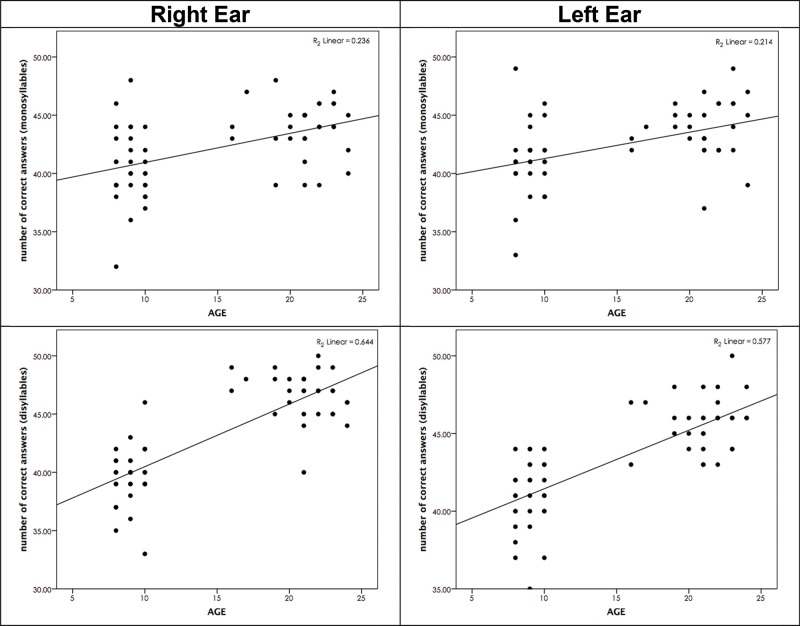
Linear regression showing the relationship between age and TCS performance per ear.

The results of our study demonstrate that young adults perform significantly better than children on the TCST in both the monosyllable and disyllable modalities and in both ears. These results were corroborated by the regression analysis, which confirmed the participation of the ‘age' variable on test performance (Figure 3).

These findings corroborate those of Keith 8, who considered the TCST to be a speech test that is part of a sub-group of tests evaluating the neuromaturational level.

Furthermore, this study demonstrated a greater effect of the age variable in the disyllable modality (64.4% for the right ear and 57.7% for the left ear) than in the monosyllable modality (23.6% for the right ear and 21.4% for the left ear).

Although phonemic discrimination is crucial in the performance of perception and speech recognition, various cognitive and linguistic processes are also activated in this task. Whether the words used in a test are more or less frequent in language can also help or hinder their recognition 15. These mechanisms may explain our results, as age accounts for more than 50% of the performance variations obtained in the disyllable modality.

According to the literature, when a large proportion of an acoustic signal is missing, the test becomes cognitive, as the individual must infer the complete message when a part is missing 8.

Because the age variable and therefore the contribution of lexical and cognitive processes seemed to affect TCST performance variations much less in the monosyllable modality than in the disyllable modality, we believe that the monosyllable modality is better for the evaluation of auditory processing, especially when evaluating adults.

This study demonstrated the effect of age (maturation factor) on performance in a time-compressed speech test. This study highlights the importance of establishing standards of normality for various age groups to make a reliable and standardized tool available for evaluating auditory closure ability in the battery of tests used to evaluate auditory processing in Brazil. We suggest that in future studies, to establish normality patterns, schooling and socioeconomic level are considered.

## AUTHOR CONTRIBUTIONS

Rabelo CM was responsible for the proposition of the original idea, data collection, data analysis, manuscript writing and review. Rocha-Muniz CN was responsible for the proposition of the original idea, data analysis, literature review, manuscript writing and review. Schochat E was responsible for the proposition of the original idea, coordination of the study, and manuscript revision.

## Figures and Tables

**Table 1:** Descriptive analysis of the performance obtained by the group of children and adults in TFC, both for the monosyllabic and the disyllabic modality.

		Mean	Standard Deviation	Minimum	Maximum	*p*-value
Monosyllables RE	Children	40.53	3.14	32.00	48.00	<0.001
	Adults	43.83	2.38	39.00	48.00	
Monosyllables LE	Children	40.70	2.65	33.00	46.00	<0.001
	Adults	43.80	2.44	37.00	49.00	
Dissyllables RE	Children	39.67	2.50	33.00	46.00	<0.001
	Adults	46.70	2.02	40.00	50.00	
Dissyllables LE	Children	40.97	2.25	35.00	44.00	<0.001
	Adults	45.70	1.74	43.00	50.00	

**Table 2:** Correlation between age and performance obtained in TCS for modalities, monosyllables and disyllables.

		Monosyllables RE	Monosyllables LE	Dissyllables RE	Dissyllables LE
Age	Pearson Correlation (r)	0.49	0.54	0.80	0.76
	*p*-value	<0.001	<0.001	<0.001	<0.001
	N	60	60	60	60
